# Aminolipids in bacterial membranes and the natural environment

**DOI:** 10.1093/ismejo/wrae229

**Published:** 2024-11-09

**Authors:** Shengwei Liu, Eleonora Silvano, Mingyu Li, Michaela Mausz, Branko Rihtman, Richard Guillonneau, Otto Geiger, David J Scanlan, Yin Chen

**Affiliations:** School of Life Sciences, University of Warwick, Gibbet Hill Road, Coventry CV4 7AL, United Kingdom; School of Life Sciences, University of Warwick, Gibbet Hill Road, Coventry CV4 7AL, United Kingdom; School of Life Sciences, University of Warwick, Gibbet Hill Road, Coventry CV4 7AL, United Kingdom; School of Life Sciences, University of Warwick, Gibbet Hill Road, Coventry CV4 7AL, United Kingdom; School of Life Sciences, University of Warwick, Gibbet Hill Road, Coventry CV4 7AL, United Kingdom; Faculty of Science and Technology, Nantes Université, CNRS, US2B, UMR 6286, Nantes F-44000, France; Centro de Ciencias Genómicas, Universidad Nacional Autónoma de México, Avenida Universidad s/n, Colonia Chamilpa, Cuernavaca, Morelos 62210, México; School of Life Sciences, University of Warwick, Gibbet Hill Road, Coventry CV4 7AL, United Kingdom; School of Biosciences, University of Birmingham, Birmingham B15 2TT, United Kingdom; Institute of Microbiology and Infection, University of Birmingham, Birmingham B15 2TT, United Kingdom

**Keywords:** aminolipids, bacterial membrane, environmental lipidomics

## Abstract

Our comprehension of membrane function has predominantly advanced through research on glycerophospholipids, also known as phosphoglycerides, which are glycerol phosphate-based lipids found across all three domains of life. However, in bacteria, a perplexing group of lipids distinct from glycerol phosphate-based ones also exists. These are amino acid-containing lipids that form an amide bond between an amino acid and a fatty acid. Subsequently, a second fatty acid becomes linked, often via the 3-hydroxy group on the first fatty acid. These amide-linked aminolipids have, as of now, been exclusively identified in bacteria. Several hydrophilic head groups have been discovered in these aminolipids including ornithine, glutamine, glycine, lysine, and more recently, a sulfur-containing non-proteinogenic amino acid cysteinolic acid. Here, we aim to review current advances in the genetics, biochemistry and function of these aminolipids as well as giving an ecological perspective. We provide evidence for their potential significance in the ecophysiology of all major microbiomes, i.e. gut, soil, and aquatic as well as highlighting their important roles in influencing biological interactions.

## Introduction

**Figure 1:**
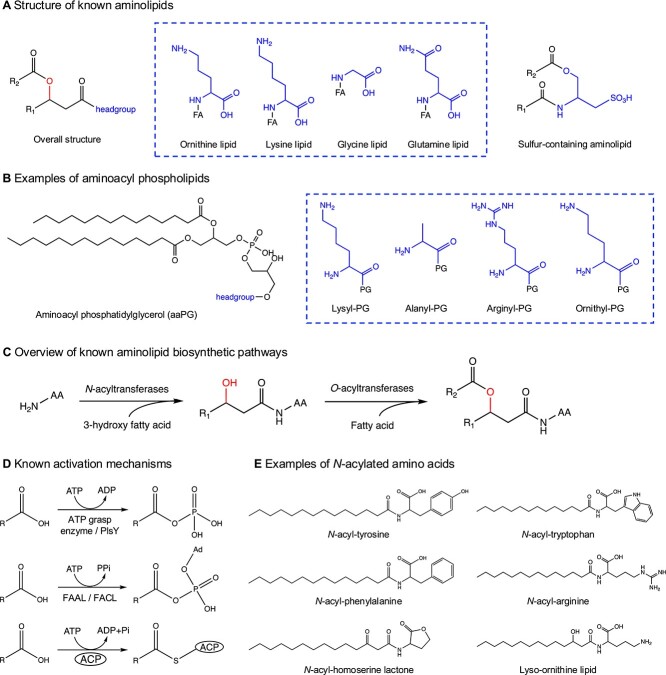
Representative structures of aminolipids and related compounds, including common fatty acid activation pathways. (A) Selected structures of several known aminolipids. (B) Examples of aminoacyl phospholipids. (C) Overview of currently known 2-step aminolipid biosynthetic pathways. (D) Currently known pathways for fatty acid activation in biological systems. (E) Selected examples of *N*-acylated amino acids, including *N*-acylhomoserine lactones and lyso-aminolipids. The 3-hydroxy group in the aminolipid is highlighted in red. FA, fatty acid chains; PG, phosphatidylglycerol; ad, adenosine; FAAL, fatty acyl-AMP ligase; FACL, fatty acyl-CoA ligase; ACP, acyl carrier protein; AA, amino acid.

Lipids comprise the building blocks for all biological membranes, providing a fluid and dynamic environment for hosting integral and peripheral membrane proteins that are critical for nutrient acquisition, defense, and cell signaling [1]. Of all lipids, glycerol phosphate based glycerophospholipids are probably the best studied given they are ubiquitous in all three domains of life. Indeed, it has been proposed that the most ancient life forms likely had a glycerophospholipid-based membrane [2]. Over recent years environmental microbiology has focused on the transformation between glycerophospholipids and non-phosphorus lipids such as betaine lipids and glycolipids [3–5]. However, a unique group of lipids which appears confined to bacteria are the amino acid containing aminolipids, often comprised of an amide linked 3-hydroxy fatty acid (R1) and an ester linked fatty acid (R2) attached to the 3-hydroxy group at R1 (Fig. 1A). These aminolipids can be integrated into both inner and outer membranes of bacterial cells [6, 7]. A noticeable structural difference between aminolipids and glycerophospholipids is that glycerophospholipids are made from a glycerol backbone whereas aminolipids are synthesized from a 3-hydroxy acyl fatty acid backbone. Several proteinogenic amino acids are found as the hydrophilic headgroup in these aminolipids, including glycine, glutamine and lysine. Non-proteinogenic amino acids have also been found including ornithine and more recently cysteinolic acid [8–10].

It is important to differentiate the aminolipids that are discussed here from aminoacyl phospholipids (Fig. 1B), the latter including lysyl-phosphatidylglycerol (PG), alanyl-PG and arginyl-PG [11]. A common feature of these aminoacyl phospholipids is to neutralize the negative charge of bacterial phospholipids (such as PG) using aminoacylated transfer RNAs (tRNAs), thus allowing pathogenic bacteria to successfully evade host immunity by preventing the action of cationic antimicrobial peptides (CAMPs). However, interested readers should look elsewhere for elegant and comprehensive reviews on amino acid decoration of phospholipids [11, 12].

## Two-step biosynthesis of aminolipids

The biosynthesis of aminolipids typically involves two steps to form the mature lipid that is composed of two fatty acid chains, although these lipids can undergo subsequent modifications such as hydroxylation, methylation and transamination. Readers interested in this topic are encouraged to refer to recent reviews for additional information [13, 14]. In the first step, fatty acids are activated before they are conjugated to an amino acid to form an amide bond (Fig. 1C). Subsequently in the second step, another fatty acid is conjugated to create the mature aminolipid (Fig. 1C). Fatty acids are generally inert chemicals and require specific biological mechanisms for activation to make them biologically available for condensation with an amino acid. Several mechanisms exist for the formation of an amide bond between fatty acids and amino acids, including acyl-phosphate, acyl-adenylate and acyl-ACP (acyl carrier protein) (Fig. 1D).

The direct condensation of ATP with a carboxylate group in fatty acids can be catalyzed by ATP grasp enzymes (Pfam 13 535) involving acyl-phosphate as the key intermediate, as seen in the formation of acyl-histidine in *Legionella pneumophila*, the causative agent of Legionnaires’ disease [15, 16]. However, it is worth noting that whether this type of system can effectively use 3-hydroxy fatty acids as a substrate has not been tested. Perhaps a more relevant example of acyl-phosphate serving as the fatty acid donor is observed in glycerophospholipid synthesis, where the PlsY enzyme transfers the acyl group from acyl-phosphate to glycerol-3-phosphate to form lyso-PG [17]. Fatty acids can also be activated to form an acyl-adenylate intermediate, e.g. by a fatty acid AMP ligase (FAAL) or a fatty acid CoA ligase (FACL) [18, 19]. However, whether FAAL/FACL enzymes can be utilized in aminolipid synthesis has yet to be established. Curiously, it appears that acyl-ACP is the donor of choice for aminolipid biosynthesis, which has been experimentally confirmed in the synthesis of ornithine-containing aminolipids [14, 20].

It is important to highlight that 3-hydroxy fatty acids are often required in the final step of ester bond formation for attaching the second fatty acid in several aminolipids (Fig. 1A). It thus appears that the aminolipid biosynthesis pathway might have “hijacked” 3-hydroxy fatty acyl-ACP from the type II fatty acid synthesis pathway, an essential intermediate in the elongation step mediated by the FabG reductase, to supply the intermediate required for aminolipid biosynthesis [21]. Similarly, several acyltransferases involved in lipid A biosynthesis, such as LpxA and LpxM, all require 3-hydroxy fatty acyl-ACP as the preferred substrate [22, 23]. Subsequent formation of lyso-aminolipids between 3-hydroxy fatty acyl-ACP and an amino acid is often carried out by an *N*-acyl transferase (Pfam 13 444), the archetype of which in ornithine lipid biosynthesis is OlsB [20].

The first step in aminolipid biosynthesis, i.e. the formation of an amide bond between the carboxylate functional group of an activated fatty acid and the amine group of an amino acid, closely resembles the biosynthesis of fatty acyl amides that are widely recognized for their role in cell signaling [24]. A large group of fatty acyl amides known as *N*-acylated amino acids, where an amino acid provides the functional amine group (Fig. 1E), are produced by a variety of organisms, including humans. Perhaps the best known of these is anandamide which is capable of binding to human cannabinoid receptors (reviewed in Ezzili et al., 2010 [25]). It is worth noting that many bacteria are also able to produce *N*-acylated amino acids although their *in vivo* function is poorly understood [26–28]. In these microbes, *N*-acylated amino acids can be synthesized through a group of GNAT acyltransferases that show some similarity to enzymes involved in aminolipid biosynthesis (see below). However, as it stands, it has not been reported whether these *N*-acylated amino acids can be further acylated to produce an aminolipid-like molecule that can be incorporated into microbial membranes. These *N*-acylated amino acids identified from soil microorganisms often do not have a 3-hydroxygroup which can be readily acylated further to form a mature membrane lipid.

**Figure 2:**
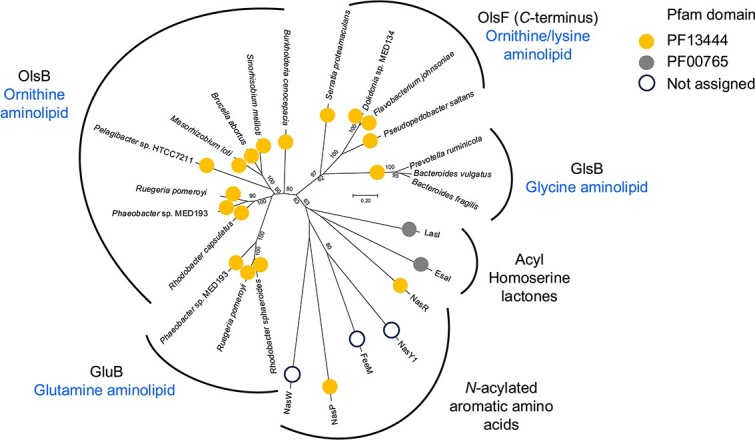
The phylogeny of *N*-acyltransferases involved in aminolipid biosynthesis together with those involved in homoserine lactone synthesis and *N*-acylated aromatic amino acid synthesis. The evolutionary history was inferred using a neighbor joining algorithm from a multiple sequence alignment obtained with ClustalX. Bootstrap values greater than 50% are shown. A total of 304 positions were used in the final dataset. Evolutionary analysis was conducted in MEGA7. Protein domain analysis was obtained from the InterProScan at EBI (https://www.ebi.ac.uk/interpro/search/sequence/). Sequences included in this analysis include OlsB (BAB1_0147 *Brucella abortus*; SMc01127 *Sinorhizobium meliloti*; SPO1980 *Ruegeria pomeroyi*; MED193_03912 *Phaeobacter* sp. MED193; BCAL1281 *Burkholderia cenocepacia*; mlr3216 *Mesorhizobium loti*; HTCC7211_00011000 *Pelagibacter* sp. HTCC7211; *Rhodobacter capsulatus*); GluB (SPO2489 *R. pomeroyi*; MED193_22491 *Phaeobacter* sp. MED193; LAZ29_20365 *Rhodobacter sphaeroides* [29]), GlsB (locus tags – BVU_RS07720 *Bacteroides vulgatus*; BM023_RS02715 *Prevotella ruminicola*; HMPREF1203_RS01905 *Bacteroides fragilis* [30]), OlsF (Spro_2569 *Serratia proteamaculans*; MED134_08291 *Dokdonia* sp. MED134; PEDSA_2277 *Pseudopedobacter saltans*; Fjoh_0833 *Flavobacterium johnsoniae* [31]), LasI (PDB 1RO5) and EsaI (PDB 1KZF) involved in SAM-dependent homoserine lactone synthesis [32, 33], and FeeM (PDB 2G0B), NasW (AAO64420), NasP (ABB76600), NasY1 (AAG53691), and NasR (AAO64421) involved in *N*-acylated amino acid synthesis [28, 34].

Phylogenetically, *N*-acyltransferases involved in aminolipid biosynthesis form a separate branch distinct from other acyltransferases, most noticeably *N*-acylated amino acid synthase and acyl-homoserine lactone synthase (Fig. 2). Domain analysis suggests that all enzymes involved in aminolipid biosynthesis (see below), along with some involved in *N*-acyl amino amide synthesis, share the common PF13444 domain. Both *N-*acylated amino acid synthase and acyl-homoserine lactone synthase use acyl-ACP for the initial step of amide bond formation. Curiously, enzymes catalyzing the amide bond formation between aromatic and non-aromatic amino acids are clearly separated phylogenetically. However, it remains to be investigated whether aromatic amino acids can indeed lead to the formation of mature aminolipids.

**Figure 3:**
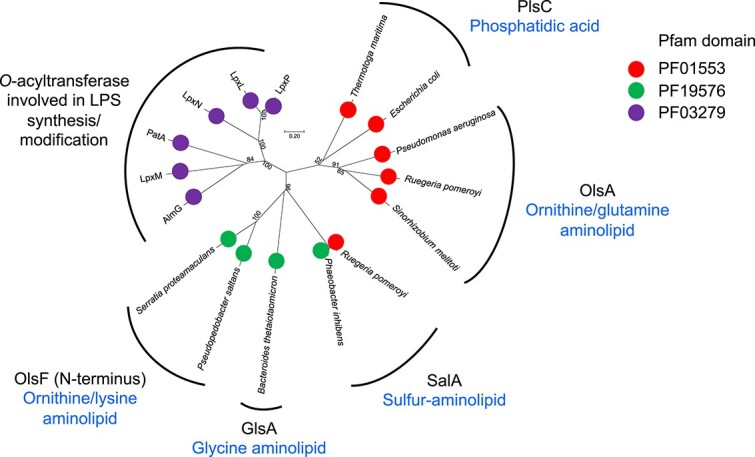
The phylogeny of *O*-acyltransferases of the lysophospholipid acyltransferase (LPLAT) family involved in the synthesis of phosphatidic acid, aminolipids as well as those involved in lipopolysaccharide (LPS) biosynthesis and modification. The evolutionary history was inferred using a neighbor joining algorithm from a multiple sequence alignment obtained with ClustalX. Bootstrap values greater than 50% are shown. A total of 557 positions were used in the final dataset. Evolutionary analysis was conducted in MEGA7. Protein domain analysis was obtained from the InterProScan at EBI (https://www.ebi.ac.uk/interpro/search/sequence/). Sequences included in this analysis include LpxP (NP_416879), LpxL (NP_415572), LpxN (NP_229869), PatA (PDB 5F2Z, [35]), LpxM (PDB 5KN7, [23]), AlmG (NP_231217, [36]), PlsC (PDB 5KYM, [37]), PlsC (NC_011748), OlsA from *Pseudomonas aeruginosa* (PA4351, [38]), *Ruegeria pomeroyi* (SPO1979, [29]), *Sinorhizobium meliloti* (SMc01116, [39]), SalA from *R. pomeroyi* (SPO0716, [8]) and *Phaeobacter inhibens* (PGA1_c01210), GlsA (BVU_RSO7715, [30]), and OlsF from *Serratia proteamaculans* (Spro_2569), *Pseudopedobacter saltens* (PEDSA_2277).

The second step in aminolipid biosynthesis typically involves an *O*-acyltransferase (Fig. 3), which shares some sequence similarity to the PlsC enzyme responsible for glycerophospholipid phosphatidic acid biosynthesis [39]. This family of *O*-acyltransferases also contains several enzymes involved in lipopolysaccharide (LPS) synthesis and modification, as well as the PatA acyltransferase involved in modifying membrane lipids in mycobacteria. Together, these proteins form a large group known as the lysophospholipid acyltransferase (LPLAT) family. Thus, it is likely that different functional domains are capable of *O*-acylation in the second step of aminolipid synthesis in different organisms and there may be new sequence motifs yet to be discovered. This consideration is important when interpreting omics-data in the context of aminolipid biosynthesis (see below).

## Known aminolipids: their synthesis and function

Several pathways for aminolipid biosynthesis in bacteria have been studied in detail in recent years. Arguably the best example is ornithine lipid [14, 40] whose synthesis is often carried out by two genes *olsB/olsA* usually found adjacent to each other on the chromosome. OlsB is an *N*-acyltransferase that forms the amide bond and OlsA an *O*-acyltransferase that forms the ester bond. In some bacteria, particularly Bacteroidetes and Flavobacteria, one single protein encoded by *olsF* is responsible for ornithine biosynthesis. While the *C*-terminus of OlsF shows a high degree of similarity to OlsB, the *N*-terminus differs from OlsA. Ornithine lipid appears widely distributed in bacteria and previous genome analyses have found more than half of bacterial genomes appear to have pathways involved in its biosynthesis.

There has been long-standing speculation that OlsB homologues are likely responsible for the initial step in the synthesis of various other aminolipids [40]. This was indeed the case for at least two other aminolipids, the glutamine lipids found in marine roseobacter clade bacteria that are important players in oceanic biogeochemical cycling [29], as well as glycine lipids found in an important group of gut microbes [30]. It is unfortunate that these two independent studies, published in the same year, both designated the *N*-acyltransferase involved in glutamine and glycine aminolipid biosynthesis as GlsB. Given this may lead to confusion in the literature we thus propose to rename the *N*-acyltransferase involved in glutamine synthesis as GluB to avoid confusion in the future. Intriguingly, these lipids appear to have a relatively restricted distribution, as determined through genome analysis. GluB is predominantly found in marine roseobacters (Alphaproteobacteria) [29] whilst GlsB is prevalent in gut associated Bacteroides [30]. The second step of the synthesis of glutamine and glycine lipids involves an *O*-acyltransferase similar to that known for the synthesis of ornithine lipids. Glycine aminolipid and its lyso form have been identified in previous omics studies of the gut microbiome [41, 42]. Indeed, the discovery of GlsB was instrumental in unveiling the synthesis of these microbiota-derived lipids for the first time. Phylogenetic analysis indicates that *gluB* genes may have arisen from a gene duplication event involving *olsB*, whereas *glsB* genes could potentially have emerged from the duplication of the C-terminus of OlsF (Fig. 2).

More recently, a new sulfur-containing aminolipid (SAL) was discovered in marine bacteria named SAL [8]. This lipid comprises a non-proteinogenic amino acid containing sulfur, cysteinolic acid. The first synthesis step, however, does not appear to be carried out by an OlsB *N*-acyltransferase homolog and the gene responsible for this step is still awaiting identification. However, the final step of *O*-acylation is performed by the *salA* gene which is homologous to *olsA*. Both OlsA and SalA share common sequence motifs with PlsC [8, 28]. However, it is worth noting that the *N*-terminus of OlsF involved in ornithine lipid biosynthesis in some flavobacteria and *Serratia* spp. lacks sequence similarity with PlsC [31]. Indeed, the OlsA/PlsC proteins feature a characteristic Pfam 01553 domain, whereas the *N*-terminus of OlsF and recently discovered *O*-acyltransferase GlyA have a different Pfam 19 576 domain (Fig. 3). Previous genome analysis has shown that *salA* appears to be confined to the marine roseobacter clade for reasons that are unclear [8]. Perhaps this restriction is related to the unique ability of these bacteria to produce and metabolize a range of C_3_ sulfonates [43, 44], including cysteinolic acid which forms the hydrophilic head group of this unusual lipid [9].

Another lesser-known aminolipid features lysine as the headgroup. This lipid, along with its hydroxylated forms, have been found in the soil bacterium *Pseudopedobacter saltans* as well as *Agrobacterium* spp. and *Pedobacter* spp. [45, 46]. However, the exact synthesis mechanism remains unclear although it is speculated that an OlsF homolog is likely involved [31, 46]. Similar to the hydroxylation of ornithine lipid by OlsE, it is hypothesized that an OlsE homolog is responsible for lysine lipid hydroxylation [46]. An OlsE homolog is also found in some marine roseobacters, such as SPO0328 in *Ruegeria pomeroyi* DSS-3 (i.d. 35%, e-value e^−54^), but it remains to be seen whether this gene is responsible for hydroxylation of the ornithine lipid or the SAL lipid found in this bacterium [8].

Unlike glycerophospholipids, the physiological functions of these aminolipids are generally not well understood and no clear pattern has yet emerged for these lipids. Indeed, it appears that in different bacteria their roles are noticeably dissimilar. For example, in the soil bacterium *Sinorhizobium meliloti*, the production of ornithine lipids is believed to be at least in part a response to phosphorus (P) deficiency. However, this transition from phospholipids to ornithine lipids does not appear to be relevant for root nodulation on legume host plants [39]. In *Pseudomonas aeruginosa*, ornithine lipid production is also enhanced under P limitation, and this lipid seems to play a role in the resistance of *Pseudomonas aeruginosa* to antimicrobial peptides [47]. However, ornithine lipids are not tightly regulated by P availability in other bacteria, e.g. *Burkholderia* sp. [48] and *Ruegeria* sp. [29]. Thus, the specific role of these lipids may differ significantly depending on the organism under investigation.

The function of glycine-containing aminolipids has been primarily studied in the context of infection and immunity, particularly in the gut microbiome (recently reviewed by Ryan et al., [49]) although many environmental bacteria are also known to produce them [10]. Both glycine and ornithine aminolipids are known to induce an immune response, but through different pathways. For example, synthetic ornithine lipids composed of saturated C14:0 fatty acids are able to bind to Toll like receptor 4 (TLR4), which is the best-known receptor for lipopolysaccharides (LPS) of Gram-negative bacteria, thus reducing the LPS-induced immune response [50, 51]. In contrast, glycine lipid such as flavolipin interacts with TLR2, a receptor known to interact with lipopeptides [51, 52]. The lyso form of flavolipin (mono-acylated) activates G-protein coupled receptors (GPCR) through internal signaling cascades [42]. Lyso glycine aminolipids display hemolytic activity and can lyse membranes [53]. This membrane-disrupting activity is similar to that of *N*-acylated amino acids that are able to disrupt bacterial membranes and exhibit antimicrobial activity [27].

In the marine environment, two unusual aminolipids, SAL and the glutamine-containing aminolipid, are largely confined to the marine roseobacter group [8, 29]. These bacteria are well known for their metabolic versatility and their ability to form various interactions with algae, marine animals and other organisms [54]. As such, we have hypothesized that these aminolipids in marine roseobacter may have a role in cell–cell interactions. This has been partially validated. For example, changes in membrane lipid composition in roseobacters seems to influence the dynamics of bacteria-protist interactions [55]. However, unlike the formation of betaine-containing lipids through membrane lipid remodeling [55], the absence of SAL lipids does not appear to affect grazing of *R. pomeroyi* DSS-3 by *Uronema* sp. (unpublished data). The loss of two aminolipids, ornithine and glutamine lipids in *R. pomeroyi* DSS-3, however, significantly alters membrane protein composition and completely changes the dynamics of bacteriophage attachment [6]. These studies suggest that the physiology of lipids is likely influenced by the interplay between lipids and integral or peripheral membrane proteins, and possibly other macromolecules that are attached to the lipid membrane, e.g. LPS. Perhaps, it is this uncertainty regarding how alterations in lipid composition can impact local protein assembly and function that makes predicting the function of aminolipids in various organisms somewhat challenging.

## Uncovering aminolipid biosynthesis in the environment through lipidomics and metagenomics

Environmental lipidomics (a.k.a. metalipidomics) has emerged as a potent tool for revealing the vast array of membrane lipids within various environmental settings, and elucidating how the environment can influence microbiome dynamics across diverse climate scenarios [56]. Nonetheless, it is puzzling that only a few studies have documented the detection of aminolipids in environmental samples through mass spectrometry-based lipidomics investigations, despite genomic analyses indicating the abundance of genes involved in aminolipid biosynthesis across many bacterial genomes. Only a handful of studies have reported the presence of ornithine aminolipids or its methylated derivatives [57–62]. This scarcity of aminolipid reports in an environmental context may be partially attributed to the insufficient utilization of extensive mass spectrometry fragmentation analyses, essential for assigning environmental lipidomics data (comprising intact lipid *m/z* and MS^n^ fragmentation patterns) to distinct aminolipid species. It is perhaps unsurprising that ornithine lipids are comparatively better documented, as a diagnostic *m/z* 115 fragment originating from dehydrated cyclized ornithine amino acid is observed in positive-mode electrospray ionization mass spectrometry [63], facilitating the identification of this aminolipid in environmental metalipidomics studies. The identification of aminolipids heavily relies on the availability of reference ion component spectra, underscoring the urgent need for further research to discover and catalogue novel aminolipid species. Current lipid databases, e.g. the Global Natural Product Social Molecular Networking (GNPS) library [64] and the LIPID MAPS Structure Database [65], are deficient in comprehensive aminolipid references, limiting their use in structural annotation of aminolipids. Employing advanced techniques such as fragment network analysis through platforms such as the GNPS could help characterization of aminolipids in future environmental lipidomics studies [59].

**Figure 4:**
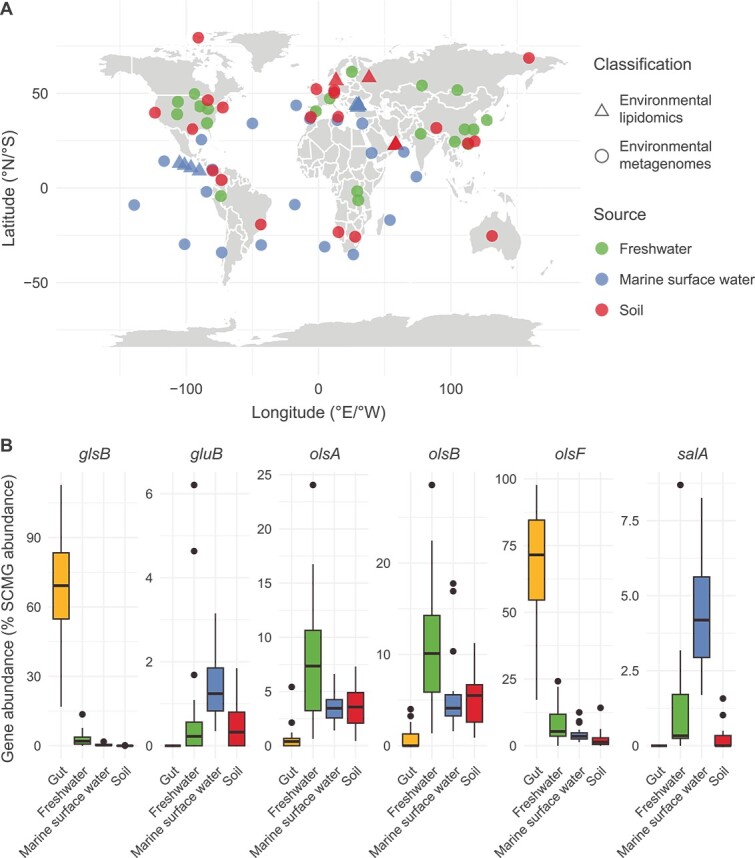
The global distribution of microbial aminolipid biosynthesis potential. (A) Detection of aminolipids through environmental lipidomics surveys, represented by triangles, and the global distribution of key genes involved in aminolipid biosynthesis in representative environmental microbiomes, including freshwater (n = 20), marine surface water (n = 20) and soil (n = 20) represented by green, blue and red circles, respectively. The environmental lipidomics dataset included in this figure is shown in Supplementary Table S1. Globally distributed metagenome datasets were retrieved from the NCBI, and basic sample and quality information is presented in Supplementary Table S2. (B) The relative abundance of aminolipid biosynthesis genes across different ecosystems, expressed as a percentage of whole-community single-copy marker gene (SCMG) abundances. Gut microbiome metagenome datasets (n = 20), with human DNA sequences removed using BMTagger, were obtained from the human microbiome project [67] accessible at https://www.hmpdacc.org/HMASM/. Briefly, raw data was trimmed to clean the data using Trimmomatic v0.39 [68], which was then used to assemble contigs using MEGAHIT v1.2.9 [69] with a *k*-mer range from 21 to 119. Gene predictions for contigs were made by prodigal v2.6.3 [70]. The aminolipid biosynthesis genes and ten bacterial single-copy housekeeping genes were searched by HMMER v3.4 [71], applying a optimized cut-off (*e*-value <1e-40). Relative gene abundances were calculated as reads per kilobase per million mapped reads (RPKM) using CoverM v0.7.0 [72]. Then, the relative abundance of aminolipid biosynthesis genes was normalized by dividing by the median abundance of ten bacterial SCMGs [73]. The taxonomy of these aminolipid biosynthesis genes was affiliated against the IMG’s unrestricted isolate genomes (IUIG) dataset (dated 11/20/2023) using DIAMOND blastp v2.1.9.163 [74] with an *e*-value <1e-5. These data are summarized in Supplementary Table S3.

It is noteworthy that the precursor for aminolipid synthesis, 3-hydroxy fatty acids, is also commonly employed as a biomarker indicative of the presence of bacterial LPS [66], which has been extensively used in paleoclimate research. Comprehensive soil, sediment, and aquatic environment samplings have revealed the widespread occurrence of 3-hydroxy fatty acids across global ecosystems. However, whether or not these molecules are truly derived from bacterial LPS is questionable, given that aminolipids are also likely prevalent in the environment. Despite the limited number of studies measuring aminolipids in the environment, recent advances in uncovering genes involved in aminolipid biosynthesis have facilitated analysis of various environmental metagenomics datasets for the presence of these genes. This analysis included representative microbiomes from marine surface water, freshwater, and soil ecosystems, as well as the human gut. The data presented in Fig. 4 reveal intriguing patterns. Whilst genes responsible for ornithine lipid biosynthesis appear ubiquitous across all ecosystems studied, the distribution of other aminolipids seems confined to specific ecosystems. For instance, the *salA* gene responsible for the synthesis of the SAL lipid is particularly abundant in marine surface water microbiomes, whereas the glycine lipid biosynthesis gene *glsB* is most prevalent in the human gut. Moreover, genes like *gluB* (required for glutamine lipid biosynthesis) and *salA* which are relatively abundant in marine bacteria, are completely absent in the human gut.

## A perspective on future research needs

Aminolipids represent an intriguing group of bacterial membrane lipids but we are only just starting to understand their physiological and metabolic roles. Activation of (3-hydroxy) fatty acids through the common bacterial type II fatty acid synthesis pathway enables subsequent adding of the 2nd fatty acid through an ester bond in a piggy-back manner. There it appears that bacteria hijacked fatty acyl-ACP from the type II fatty acid synthesis pathway to make aminolipids. Given the type II fatty acid synthesis pathway is largely found in bacteria, this could help explain the lack of these lipids in archaea and eukaryotes. However, given that the synthesis of the sulfur-containing aminolipids SAL in roseobacters does not require a 3-hydroxy fatty acid, it thus remains to be seen whether similar aminolipids may exist beyond bacteria.

To date, only a handful of amino acids are found in these lipids and questions remain whether other aminolipids exist in bacterial membranes. Noticeably, to date no aromatic amino acids are found in aminolipids (e.g. tyrosine, phenylamine), nor are amino acids with heterocyclic rings found (e.g. histidine, proline) yet their *N*-acyl amino acid counterparts appear common in the biosphere. To address these disparities, a thorough, systematic and meticulous investigation of membrane lipids in a wide range of bacterial isolates and model organisms using high-resolution mass spectrometry is clearly required in future lipidomics studies. For some aminolipids that are known to be present in bacterial membranes, their synthesis pathways also remain to be fully established, e.g. lysine aminolipids and SALs. Uncovering the genes underpinning their biosynthesis would greatly advance our understanding of the biosynthetic potential of particular microbiomes, helping guide the design of metalipidomics surveys to confirm their presence and quantify their abundance.

Functional studies of these lipids in microbial membranes are still in their infancy. Both glycine lipids and ornithine lipids, which are abundant in the human gut microbiome (Fig. 4), have been identified as potential immunomodulators. Ornithine lipids produced by the gut bacterium *Akkermansia muciniphila* likely influence the host’s production of pro-inflammatory cytokines and antimicrobial peptides through interaction with the host transcription factor ATF3 [75]. Ornithine itself can also influence gene expression by interacting with ribosomes [76]. Other aminolipids such as SAL and glutamine lipids are not as widely distributed as ornithine lipids, and their functions appear to be group or strain-specific, depending on the model organisms investigated. For example, SAL lipids in *Phaeobacter* sp. play a role in biofilm formation, yet in a closely related marine roseobacter strain *R. pomeroyi* DSS-3, deletion of the *salA* gene had little impact on biofilm formation. Thus, it is entirely possible that the interplay between lipids-lipids and lipids-proteins varies between bacterial strains, making precise prediction of aminolipid function in bacterial physiology not at all straightforward. Moreover, from a fundamental perspective, it is puzzling why bacteria encode the capacity to produce these lipids in the first place. For example, the well-studied model bacterium *Escherichia coli* does not appear to produce any aminolipids. After all, evolution has selected glycerophospholipids from the beginning of life’s existence [2]. Thus, future studies should employ a wide variety of aminolipid-producing models to investigate their roles across a range of bacteria-bacteria and bacteria-host interactions. Only through such extensive work can a pattern for the role of these lipids begin to emerge.

## Supplementary Material

Table_S1_wrae229

Table_S2_wrae229

Table_S3_wrae229

## Data Availability

There are no primary data associated with this review article.
